# *Bombyx mori* gloverin A2 alleviates enterotoxigenic *Escherichia coli*-induced inflammation and intestinal mucosa disruption

**DOI:** 10.1186/s13756-019-0651-y

**Published:** 2019-11-26

**Authors:** Qian Lin, Guoqi Su, Aimin Wu, Daiwen Chen, Bing Yu, Zhiqing Huang, Yuheng Luo, Xiangbing Mao, Ping Zheng, Jie Yu, Junqiu Luo, Jun He

**Affiliations:** 1 Institute of Animal Nutrition, Sichuan Agricultural University, Chengdu, Sichuan 611130 People’s Republic of China; 2 Key Laboratory for Animal Disease-Resistance Nutrition and Feed, Ministry of Agriculture, Chengdu, Sichuan 625014 People’s Republic of China; 3 Guangdong Key Laboratory for Innovative Development and Uilization of Forest Plant Germplasm, South China Agricultural University, Guangzhou, 510642 China

**Keywords:** BMGlvA2, *E. coli* K88, Mice, Inflammation, Intestinal mucosa

## Abstract

**Background:**

Enterotoxigenic *Escherichia coli* (ETEC) is one of the leading bacterial causes of intestinal inflammation and diarrhea. However, the ETEC is frequently resistant to common antibiotics. In this study, we explored the role of a novel antibacterial peptide *Bombyx mori* gloverin A2 (BMGlvA2) in alleviating ETEC-induced inflammation and intestinal epithelium disruption in mice.

**Methods:**

An ETEC-challenged mice model was used, and the ETEC-challenged mice and non-challenged mice were treated by the BMGlvA2 at different doses.

**Results:**

ETEC challenge not only elevated the concentrations of serum inflammatory cytokines such as the IL-6 and TNF-α (*P* < 0.01), but also elevated the concentrations of serum creatinine and urea (*P* < 0.05). However, BMGlvA2 attenuated the inflammatory responses by decreasing the serum inflammatory cytokines and improving the metabolisms in ETEC-challenged mice, and alleviated the ETEC-induced tissue damage in spleen. Moreover, BMGlvA2 treatment significantly elevated the duodenum villus height and decreased the crypt depth in the duodenum and ileum in ETEC-challenged mice (*P* < 0.05). Interestingly, BMGlvA2 improved the distribution and abundance of tight-junction protein ZO1 in duodenum and ileum epithelium after ETEC-challenge. Moreover, BMGlvA2 significantly down-regulated the expression levels of inflammatory cytokines (IL-1β, IL-6, and TNF-α) and the apoptosis-related genes (Caspase 8 and Caspase 9) in jejunal mucosa (*P* < 0.05) in the TETC-challenged mice. Importantly, BMGlvA2 significantly elevated the expression levels of critical genes related to mucosal barrier functions such as the mucins (MUC1 and MUC2) and glucose transporter (GLUT2) in the intestinal mucosa (*P* < 0.05).

**Conclusion:**

Our results suggested a novel function of the conventional antibacterial peptides, and the anti-bacterial and anti-inflammatory properties of BMGlvA2 may allow it a potential substitute for conventionally used antibiotics or drugs.

## Background

Animal diseases caused by enterotoxigenic *Escherichia coli* (ETEC) infection typically appear as severe diarrhea and rapid dehydration. Human and mammlian animals such as the mice and pigs are susceptible to ETEC [1, 2]. ETEC adhere to the small intestinal microvilli, which induces morphological lesions and produce enterotoxins acting locally on enterocytes [3]. Previous studies indicated that endotoxins usually caused intestinal mucosa inflammation and destruction of tight junction integrity and epithelial cell apoptosis [4], which subsequently led to disruption of intestinal homeostasis and damage of the intestinal barrier functions. Currently, antibiotics and other chemical drugs have been widely used to treat the ETEC infection. However, long-term or high-dose treatments of antibiotics not only lead to drug resistance, but also lead to their residues in animal product. For instance, polypeptides (colistin), aminoglycosides (gentamicin, streptomycin) and tetracyclines (Doxycycline) are generally prescribed for infections caused by gram-negative bacteria [5–8]. However, these drugs may cause various Adverse Drug Reactions (ADRs), such as allergy, diarrhea, nausea and hypopsia etc. [9, 10]. Therefore, substitute for conventionally used antibiotics has attracted considerable research interest worldwide.

The antimicrobial peptides (AMPs) are a group of immune-related peptides/ proteins that protect the host from microbial infections [11–13]. Antimicrobial peptides exhibit extensive activity against gram-positive and gram-negative bacteria, yeasts, and fungi [14]. Especially, they exhibit activity against some antibiotics-resistant bacterial species. For instance, the tridecaptin M was found to exhibit activity against many colistin-resistant *E. coli* strains in vitro and in vivo [15]. Importantly, a number of studies have shown that AMPs plays a critical role in immune regulation [16, 17]. For instance, the AMPs were found to regulate the secretion of inflammatory cytokines in a variety of animal species [18, 19]. In the last decades, a number of AMPs such as the ceropins, histatins, defensins, and cathlicidins have been isolated from various animals or microbial species [20, 21]. BMGlvA2 is an induced antimicrobial insect protein isolated from *Bombyx mori* (*B. mori*), which is a small cationic linear α-helical peptide belonging to cecropin family [22]. BMGlvA2 was found to inhibit the growth of bacteria by binding to the lipopolysaccharide (LPS) components existing on the surface of bacterial membrane, which increases the permeability of the membrane. However, it has no toxic effects on mammalian cells [22]. Although evidences are accumulating to show that the AMPs can serve as a critical regulator for diverse biological events including immune responses [23–26], the involvement of BMGlvA2 in regulating the ETEC-induced inflammation and intestinal disruption is just beginning to be explored.

In our previous study, the BMGlvA2 was successfully expressed and purified by using a heterologous system, and the recombinant BMGlvA2 was found to show a moderate antibacterial activity against the ETEC [27]. In this study, we explored the role of the BMGlvA2 in alleviating ETEC-induced inflammation and intestinal epithelium disruption in mice. Our result offers novel insights into the role of the AMPs and will also facilitate the development of novel substitute for conventionally used antibiotics or drugs.

## Methods

### Preparation of BMGlvA2

The engineered strain L Orgami B (DE3)-harboring the recombinant plasmid (pet32a-gloverin A2) was previously constructed in our laboratory [27]. The protein expression was induced by 1.0 mM isopropyl β-d-1 thiogalactoside (IPTG). After incubation for 8 h at 28 °C, bacterial cells were harvested by centrifugation at 8000 r/min for 15 min at 4 °C, 20 mL phosphate buffer solution (PBS) was added to washing the precipitation, centrifuged at 8000 r/min for 15 min at 4 C, and the supernatant was discarded. Then 20 ml lysis buffer was added and incubated at 4 °C for the overnight. Then, schizolytic cells were sonicated (4 s pulse and 8 s interval; 30 cycles; Sonics-Vibra cell, USA). The supernatant was harvested by centrifugation at 8000 r/min for 20 min at 4 °C. The supernatant obtained above was filtered by 0.22 μm filter, and then applied to Ni 2^+^ − IDA column (Sangon Biotech, China) and purified according specification. Protein concentration was quantified by the BCA assay (Beyotime, China).

### Animal trial

The experimental procedure and animal care were carried out in compliance with the regulations of the Animal Care Committee of Sichuan Agricultural University (No. 20180701). Sixty male ICR (Institute of Cancer Research) mice (4 weeks) were purchased from the Da Shuo laboratory animal Co., Ltd. (Chengdu, China). A 2 (sterile saline or *E. coli* K88 challenge) × 3 (three BMGLvA2 doses) factor design was used and mice were randomly divided into 6 treatments according to the principle of similar body weight (*n* = 10). All mice were housed at single cage at a constant humidity (40–70%) and temperature (20–25 °C) under a 12 h light/dark cycle with free access to water and feed. The injections of BMGlvA2 (0, 4, 8 mg/kg) were carried out for 6 days (once a day) via 1 ml insulin syringe (Braun, Melsungen, Germany). At 7 d, mice were either challenged (intraperitoneal injection) by 200 μl sterile saline or *E. coli* K88 culture solution (OD_600_ = 0.5). Five hours after challenge, the mice were anesthetized via 20-s exposure to carbon dioxide and subjected to collect the blood samples by cardiac puncture [28]. Duodenum, jejunum and ileum samples were taken immediately after cervical dislocation. A portion of the sample was fixed in formaldehyde solution for morphological observation and the other portion was rapidly frozen in liquid nitrogen and stored at − 80 °C until analysis. Blood samples were centrifuged at 3000×g for 15 min at 4 °C, after which the serum was separated and stored at − 20 °C for further analysis.

### Cytokine measurements

IL-1β, IL-6 and TNF-α (Beijing Sizhengbai Biotech, China), D-Lactic acid (Beijingchenglin Biotech, China) were determined by enzyme-linked immunosorbent assay (ELISA), using commercial kits according to the manufacturer’s recommendations. Albumin (ALB), total protein (TP), Urea, creatinine (CRE), c-reactive protein (CRP), aspartate transaminase (AST) and alanine aminotransferase (ALT) were detected by The 3100 type automatic biochemical analyzer (Hitachi, Tokyo, Japan), and globulin (GLB) were calculated based on TP and ALB.

### Histopathological assays

Histological analysis was performed on duodenum, jejunum, ileum, and spleen. The samples were fixed overnight in 4% paraformaldehyde and then dehydrated with different concentrations of ethanol. After dehydration, samples were embedded in paraffin and were subsequently cut into 4-μm thick sections. The prepared tissue sections were stained with hematoxylin and eosin (H&E) and sealed with a neutral gum. The image of spleen tissue sections was analyzed by using the Image-pro Plus 6.0 (Media Cybernetics, USA), and the intestinal villus height and crypt depth were measured by using an image processing and analysis system (Image-Pro Plus 6.0, Media Cybernetics, Inc., Bethesda, MD, USA).

### Immunofluorescence staining

The jejunal tissue section was deparaffinized and rinsed with distilled water for 5 min. Tissue sections were then subjected to antigen retrieval by ethylenediaminetetraacetic acid (EDTA, 1 mol/L, pH 9.0, Gooddbio Technology Co., Ltd., Wuhan, China). Before overnight incubating at 4 °C with rabbit anti-ZO-1 polyclonal antibody (Gooddbio Technology Co., Ltd., Wuhan, China), sections were blocked with 3% bovine serum albumin. The sections were washed three times with PBS (pH 7.4) for 5 min each time, and then goat anti-rabbit IgG-FITC secondary antibody (Gooddbio Technology Co., Ltd., Wuhan, China) was added thereto, followed by incubation at room temperature for 50 min in the dark. 4′,6-diamidino-2-phenylindole (DAPI, Gooddbio Technology Co., Ltd., Wuhan, China) stain was added to incubated for 10 min at room temperature after tissue sections were washed with PBS (pH = 7.4). Finally, the fluorescence of the sections was visualized by a confocal scanning microscope (NIKON ECLIPSE TI-SR), and the images were taken using NIKON DS-U3 software.

### RNA isolation and quantitative RT-PCR

Total RNA was extracted from jejunal samples using TRIzol Reagent (TaKaRa, Dalian, China). Then, each RNA sample was reverse-transcribed into cDNA using reverse transcriptase (Takara, Tokyo, Japan) after detection of RNA concentration and purity by spectrophotometer (Beckman Coulter, DU800). The PCR primer sequences were designed using Primer Premier 5.0 and are listed in Additional file 1: Table S1. Briefly, quantitative PCR was performed by QuanStudio 6 Flex Real-Time PCR detection system (Applied Biosystems, Foster City, CA, USA) with a total of 10 μL of assay solution containing 5 μL SYBR Green mix (Takara), 0.2 μL Rox, 3 μL deionized H2O, 1 μL cDNA template, and 0.4 μL each of forward and reverse primers. The comparative Ct value method was used to quantify mRNA expression relative to β-actin expression.

### Statistics analysis

All statistical analysis was performed using SPSS 21.0 software. The individual mouse was used as the experimental unit, and all data were expressed as mean ± standard error (SEM). Statistical analysis was carried out using two-way ANOVA followed by Duncan’s multiple comparisons test. Image production using GraphPad Prism software (Version 7. GraphPad Software Inc., CA, USA).

## Results

### Effect of BMGlvA2 on fecal score and integrity of immune organ

**Fig. 1:**
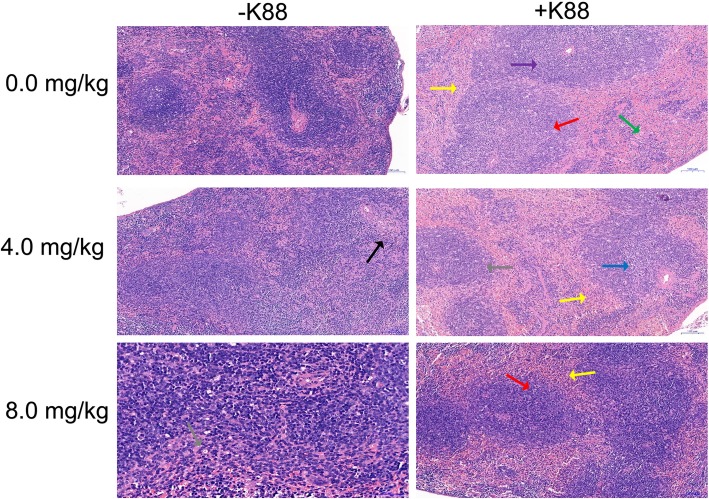
Effect of BMGlvA2 on morphology of the spleen in mice. Mice were sacrificed 5 h after injection of *E. coli* K88 or LB medium. The spleen exhibited mild focal degeneration, necrosis, and expansion of splenic nodules in the white pulp area in the ETEC-challenged mice (mice in the same group showed the same trend). Red arrows indicate splenic node expansion, black arrow indicates germinal centers are not evident, blue arrow indicates focal degeneration and necrosis of lymphocytes in the white pulp area, green arrow indicates increased macrophages, gray arrows indicate decreased lymphocytes in the germinal center, yellow arrows indicate increased neutrophils in the red pulp area, purple arrow indicates decreased lymphocytes in the white pulp area

In this study, the BMGlvA2 has been successfully expressed and purified (Additional file 1: Figure S1). Mice were challenged either with saline or ETEC after treated the purified BMGlvA2. The fecal score was analyzed by Disease activity index (DAI) [29]. We found that ETEC challenge significantly increased the fecal score. However, BMGLlvA2 injection decreased the fecal score in the ETEC-challenged mice (Table.1). Interestingly, histopathological assays showed that the spleen exhibited mild focal degeneration, necrosis, and expansion of splenic nodules in the white pulp area in the ETEC-challenged mice (mice in the same group showed the same trend). However, BMGLlvA2 attenuated the ETEC-induced tissue damage in the spleen (Fig. 1).

**Table 1:** Effect of BMGlvA2 on fecal score of the mice

-K88			+K88			*P* value		
0.0 mg/kg	4.0 mg/kg	8.0 mg/kg	0.0 mg/kg	4.0 mg/kg	8.0 mg/kg	Interaction	K88	A
0.00 ± 0.00^b^	0.00 ± 0.00^b^	0.40 ± 0.08^ab^	1.30 ± 0.16^a^	0.30 ± 0.10^ab^	0.50 ± 0.09^ab^	0.094	0.020	0.229

The data were expressed as mean ± SEM. Fecal fraction of mice within 5 h after injection of *E. coli* K88 or LB medium. The fecal score was analyzed by Disease activity index (DAI). Data with different superscript letters in a row indicated that the differences between different treatment groups were statistically significant (*p* < 0.05)

### Effect of BMGlvA2 on serum inflammatory cytokines and metabolic indicators

Some of the critical inflammatory cytokines and metabolic indicators in serum were investigated. As shown in Table 2, the serum concentrations of Urea, CREA, IL-6, and TNF-α were significantly increased in the mice upon ETEC challenge (*P* < 0.05). While the concentrations of globulin, albumin, and total protein were significantly decreased in the ETEC-challenged mice (*P* < 0.05). Interestingly, BMGlvA2 significantly reduced the serum concentrations of inflammatory cytokines such as the IL-6, and TNF-α (*P* < 0.05). Moreover, the serum concentrations of UREA and CREA were decreased in the ETEC-challenged mice upon BMGlvA2 treatment (*P* < 0.05).

**Table 2:** Effect of BMGlvA2 on inflammatory cytokines and metabolic indicators in serum

Indicators	-K88			+K88			*P* value		
	0.0 mg/kg	4.0 mg/kg	8.0 mg/kg	0.0 mg/kg	4.0 mg/kg	8.0 mg/kg	Interaction	K88	A
IL-1β ng/mL	219.90 ± 0.01	220.10 ± 0.02	220.10 ± 0.03	220.00 ± 0.04	220.10 ± 0.03	220.00 ± 0.02	0.261	0.847	0.371
IL-6 ng/mL	9.14 ± 0.1027^b^	9.47 ± 0.14^b^	9.06 ± 0.08^b^	79.25 ± 4.88^a^	28.52 ± 5.10^b^	9.54 ± 0.09^b^	< 0.0001	< 0.0001	< 0.0001
TNFα ng/mL	215.60 ± 0.07^b^	215.90 ± 0.16^b^	215.70 ± 0.08^b^	230.50 ± 1.68^a^	218.10 ± 0.70^b^	215.80 ± 0.10^b^	< 0.0001	< 0.001	< 0.001
D-LA μg/L	3.27 ± 0.00	3.28 ± 0.01	3.23 ± 0.03	3.32 ± 0.00	3.29 ± 0.01	3.28 ± 0.01	0.261	0.847	0.371
CRP mg/L	4.32 ± 0.17^ab^	3.93 ± 0.23^b^	2.67 ± 0.09^b^	4.70 ± 0.38^ab^	6.06 ± 0.55^a^	4.70 ± 0.25^ab^	0.373	0.010	0.138
TP g/L	60.50 ± 0.61^a^	58.83 ± 0.23^a^	55.52 ± 0.24^ab^	52.50 ± 0.73^b^	55.87 ± 0.62^ab^	57.43 ± 0.46^ab^	0.003	0.008	0.721
GLO g/L	28.47 ± 0.35^a^	27.67 ± 0.17^a^	27.10 ± 0.29^ab^	24.82 ± 0.45^b^	25.88 ± 0.39^b^	27.45 ± 0.29^a^	0.069	0.018	0.724
ALB g/L	32.03 ± 0.28^a^	31.27 ± 0.10^a^	29.52 ± 0.25^ab^	27.68 ± 0.31^b^	29.98 ± 0.27^ab^	29.98 ± 0.22^ab^	0.001	0.001	0.296
A/G	1.13 ± 0.01^ab^	1.13 ± 0.01^ab^	1.09 ± 0.01^b^	1.12 ± 0.012^ab^	1.16 ± 0.01^a^	1.09 ± 0.01^b^	0.654	0.681	0.076
UREA mmol/L	8.07 ± 0.11^b^	7.81 ± 0.12	7.45 ± 0.07^b^	26.24 ± 1.66^a^	7.10 ± 0.09^b^	8.99 ± 0.07^b^	< 0.0001	0.0004	< 0.0001
CREA μmol/L	6.00 ± 0.23^b^	6.48 ± 0.19^ab^	7.09 ± 0.19^ab^	26.25 ± 2.76^a^	6.65 ± 0.13^ab^	7.08 ± 0.21^ab^	0.182	0.113	0.519
ALT U/L	39.83 ± 1.63^a^	24.40 ± 0.41^c^	18.60 ± 1.85^c^	36.33 ± 2.91^ab^	27.17 ± 0.99^bc^	24.60 ± 0.39^c^	0.510	0.609	0.001
AST U/L	303.50 ± 25.74^a^	147.00 ± 6.33^b^	174.30 ± 18.02^b^	325.00 ± 26.54^a^	131.80 ± 7.45^b^	249.70 ± 20.66^ab^	0.596	0.466	0.002

The data were expressed as mean ± SEM. Data with different superscript letters in a row indicated that the differences between different treatment groups were statistically significant (*p* < 0.05). *IL-1β* interleukin 1 beta, *IL-6* interleukin 6, *TNFα* tumor necrosis factor alpha, *D-LA* d-lactic acid, *CRP* c-reactive protein, *TP* total protein, *GLO* globulin, *ALB* albumin, *A/G* albumin/globulin, *CREA* creatinine, *ALT* alanine aminotransferase, *AST* glutinous straw transaminase

### Effect of BMGlvA2 on intestinal morphology and the distribution of zonula occludens-1 proteins

**Fig. 2:**
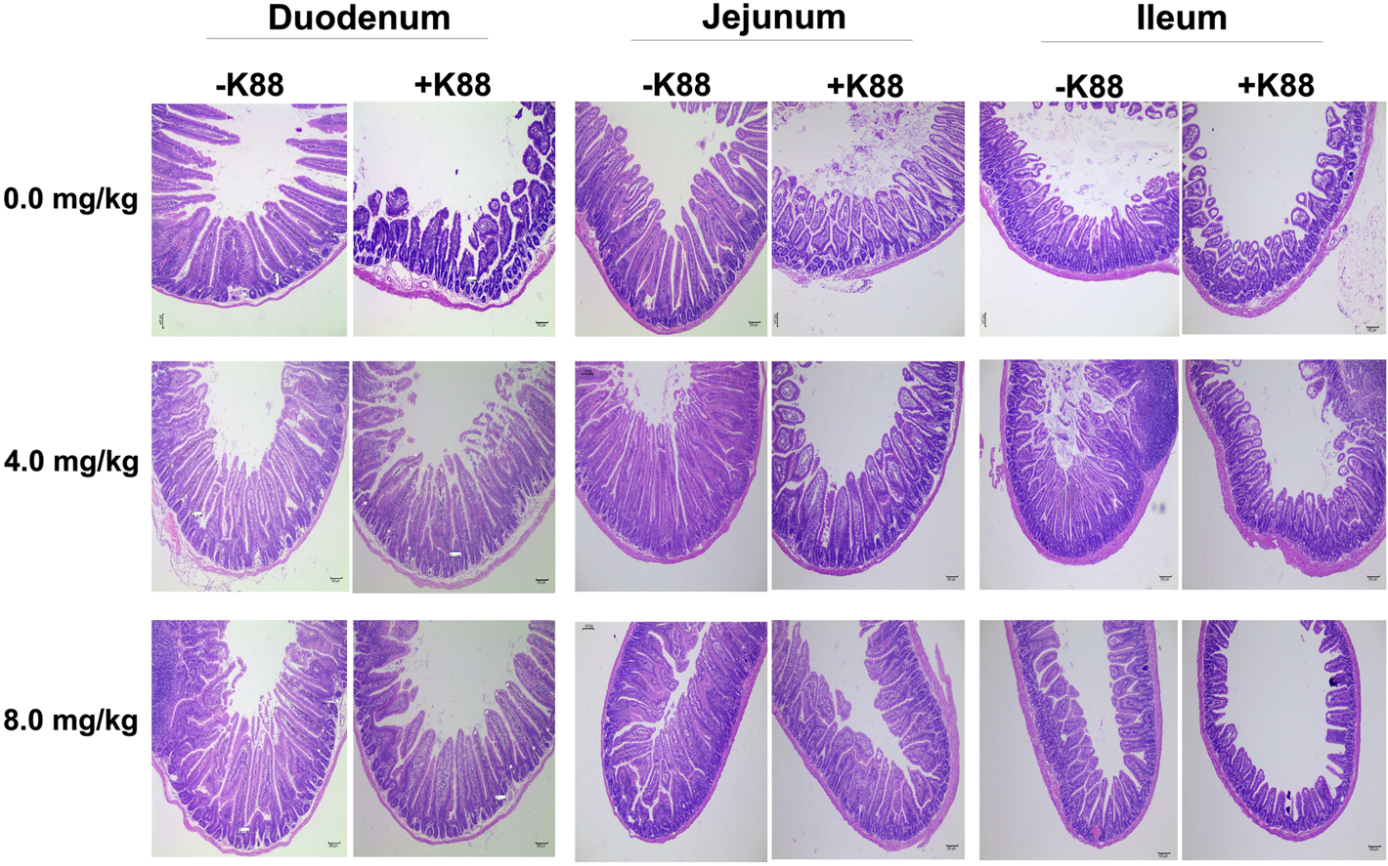
Effect of BMGlvA2 on the morphology of the intestine. Mice were sacrificed 5 h after injection of *E. coli* K88 or LB medium. Duodenum, jejunum and ileum were surgically removed, fixed, stained with H&E, and microscopically examined (mice in the same group showed the same trend)

**Fig. 3:**
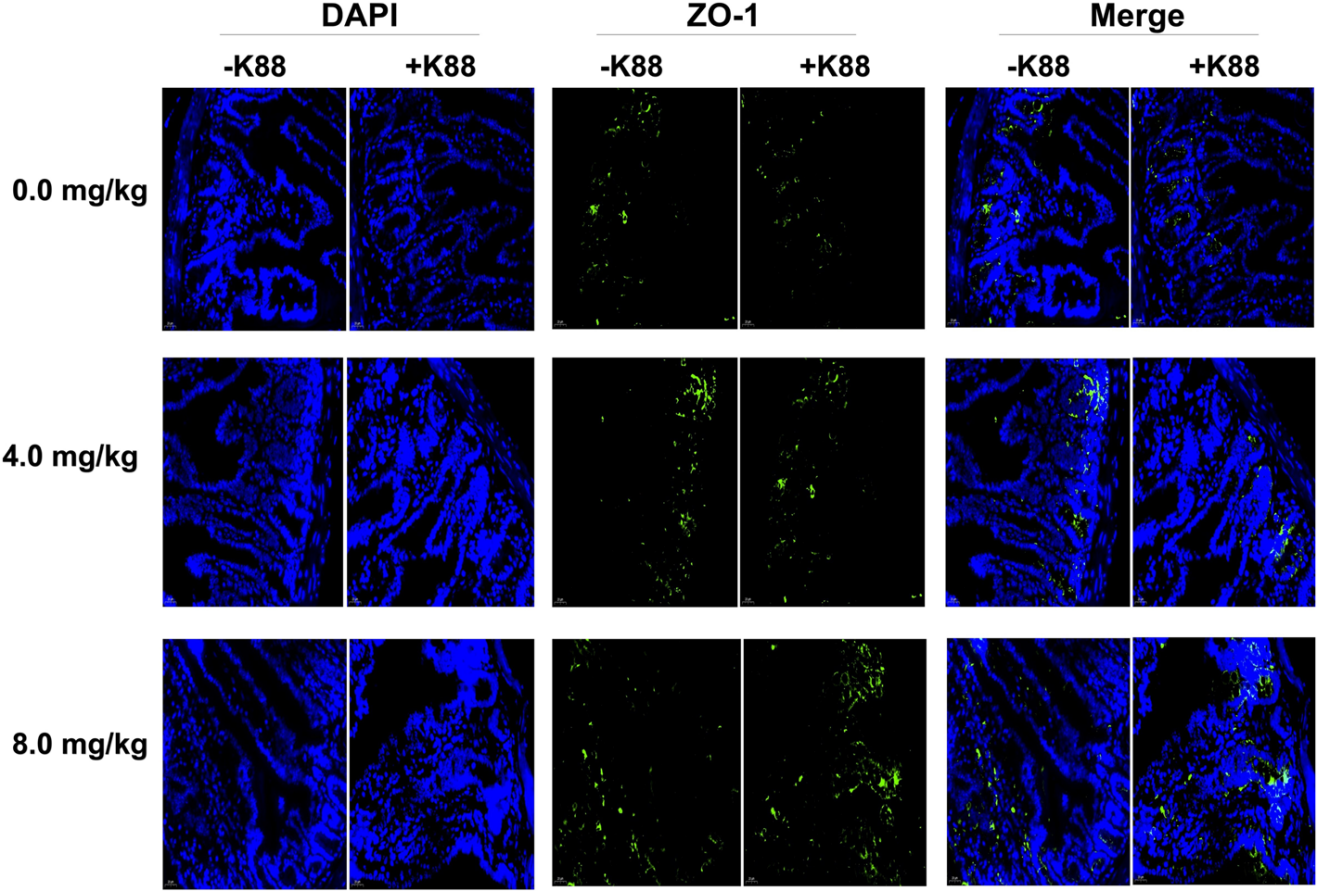
Effect of BMGlvA2 on distribution and abundance of ZO1 proteins. Representative immunofluorescent images for detection of ZO1 (green) and DAPI (blue). Scale bar = 20 μm

Histopathological assays indicated that ETEC-challenge impaired the intestinal mucosa, which has been evidenced by shortened villi, necrosis, and loss of epithelial cells in the intestinal epithelium (Fig. 2). After quantitative analysis, we found that ETEC-challenge significantly decreased the villus height in the duodenum and jejunum (*P* < 0.05). Moreover, ETEC-challenge significantly increased the crypt depth and decreased the ratio of villus height to crypt depth (V/C) in the ileum (Table 3). However, BMGlvA2 treatment at a high dose (8 mg/kg) attenuated the ETEC-induced mucosa lesion. The villus height of duodenum in the BMGlvA2-treated mice (8 mg/kg) was higher than the ETEC-challenged mice (*P* < 0.05). Moreover, BMGlvA2 treatments at a higher dose (8 mg/kg) decreased the crypt depth both in the duodenum and ileum (*P* < 0.05). Additionally, we explored the distribution and abundance of zonula occludens-1 (ZO-1), one of the major tight-junction-related proteins located in the intestinal epithelium, by immunofluorescence analysis. We found that the ZO-1 staining in the jejunum was diffuse with little staining at the intercellular tight junction region in the ETEC-challenged mice, indicating disruption of the tight junction upon ETEC infection (mice in the same group showed the same trend). However, BMGlvA2 treatment attenuated the ETEC-induced disruption by improving the localization and abundance of the ZO1 proteins in the intestinal epithelium (Fig. 3).

**Table 3:** Effect of BMGlvA2 on intestinal morphology

Organization	Indicators	-K88			+K88			*P* value		
		0.0 mg/kg	4.0 mg/kg	8.0 mg/kg	0.0 mg/kg	4.0 mg/kg	8.0 mg/kg	Interaction	K88	A
Duodenum	Villus height (μm)	420.14 ± 8.40^a^	434.40 ± 14.82^a^	417.23 ± 6.45^a^	317.27 ± 6.89^c^	394.78 ± 8.47^ab^	343.29 ± 6.51^b^	0.374	< 0.0001	0.117
	Crypt depth (μm)	106.59 ± 2.35^ab^	99.25 ± 2.28^ab^	93.80 ± 1.89^b^	115.07 ± 2.52^a^	116.02 ± 4.49^a^	81.47 ± 1.21^c^	0.086	0.424	0.002
	V/C	3.10 ± 0.12	4.36 ± 0.09	4.50 ± 0.11	2.77 ± 0.04	3.57 ± 0.16	4.22 ± 0.07	0.183	0.001	0.002
Jejunum	Villus height (μm)	211.80 ± 2.34^ab^	231.49 ± 11.34^a^	195.86 ± 4.34^ab^	172.00 ± 5.217^b^	179.65 ± 9.01^b^	181.40 ± 3.99^b^	0.526	0.014	0.565
	Crypt depth (μm)	91.64 ± 3.49	90.52 ± 3.20	75.90 ± 2.14	79.59 ± 2.49	77.33 ± 3.37	74.45 ± 3.78	0.703	0.166	0.356
	V/C	2.39 ± 0.08	2.54 ± 0.08	2.63 ± 0.07	2.17 ± 0.03	2.31 ± 0.05	2.57 ± 0.10	0.850	0.250	0.217
Ileum	Villus height (μm)	331.30 ± 18.31	352.29 ± 14.47	350.19 ± 6.04	319.60 ± 6.21	235.76 ± 13.57	332.22 ± 14.50	0.986	0.671	0.779
	Crypt depth (μm)	91.40 ± 2.84^b^	102.19 ± 3.72^ab^	92.05 ± 0.99^b^	123.23 ± 3.56^a^	103.59 ± 5.33^ab^	94.09 ± 2.38^b^	0.125	0.090	0.241
	V/C	3.66 ± 0.20^a^	3.46 ± 0.10^ab^	3.81 ± 0.068^a^	2.61 ± 0.07^b^	3.38 ± 0.05^ab^	3.51 ± 0.10^ab^	0.245	0.063	0.245

The data were expressed as mean ± SEM. Data with different superscript letters in a row indicated that the differences between different treatment groups were statistically significant (*p* < 0.05).V/C, the ratio of villus height to crypt depth

### Effect of BMGlvA2 on inflammatory response genes in the intestinal epithelium

**Fig. 4:**
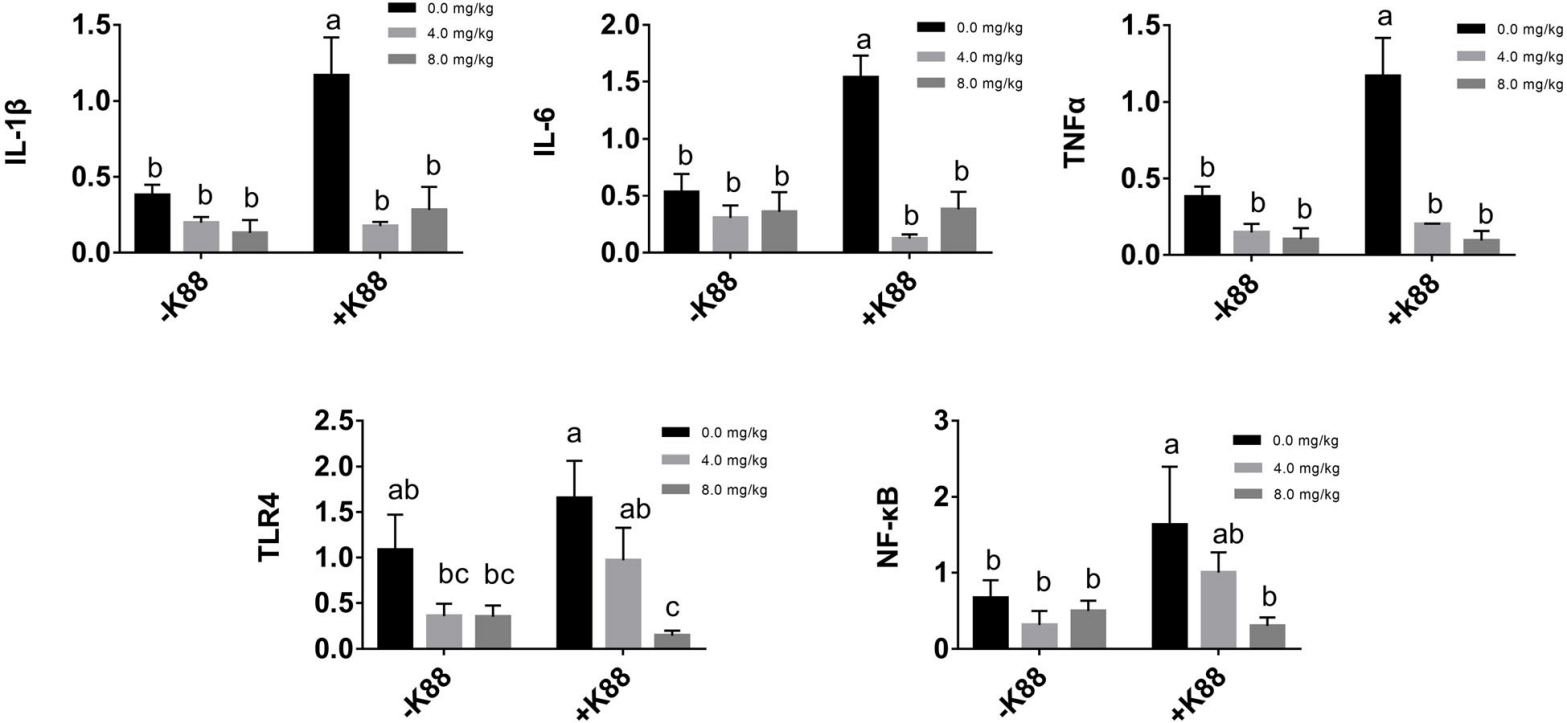
Effect of BMGlvA2 on the expression of genes related to inflammatory responses. Total RNA was extracted from jejunum tissue and the expression of related genes in jejunum was measured by real-time fluorescence PCR. The target gene mRNA expression level was calculated using the 2^–ΔΔCt^ method. ^a-c^ Values within a column differ if they do not share a common superscript (*P* < 0.05). IL-1β, interleukin 1 beta; IL-6, interleukin 6; TNFα, tumor necrosis factor alpha; TLR4: toll-like receptor 4; NF-κB: nuclear factor-kappa B

As shown in Fig. 4, ETEC challenge significantly elevated the expression levels of inflammatory response genes such as the IL-1β, IL-6, and TNF-α in the intestine (*P* < 0.05). However, BMGlvA2 treatment down-regulated their expression levels in the ETEC-challenged mice (*P* < 0.05). Moreover, ETEC challenge activated the expression of two critical molecules (TLR4 and NF-κB) related to inflammation signaling pathway. Interestingly, BMGlvA2 treatment at a high dose (8 mg/kg) significantly decreased their expression levels (*P* < 0.05).

### Effect of BMGlvA2 on critical genes related to intestinal barrier functions

**Fig. 5:**
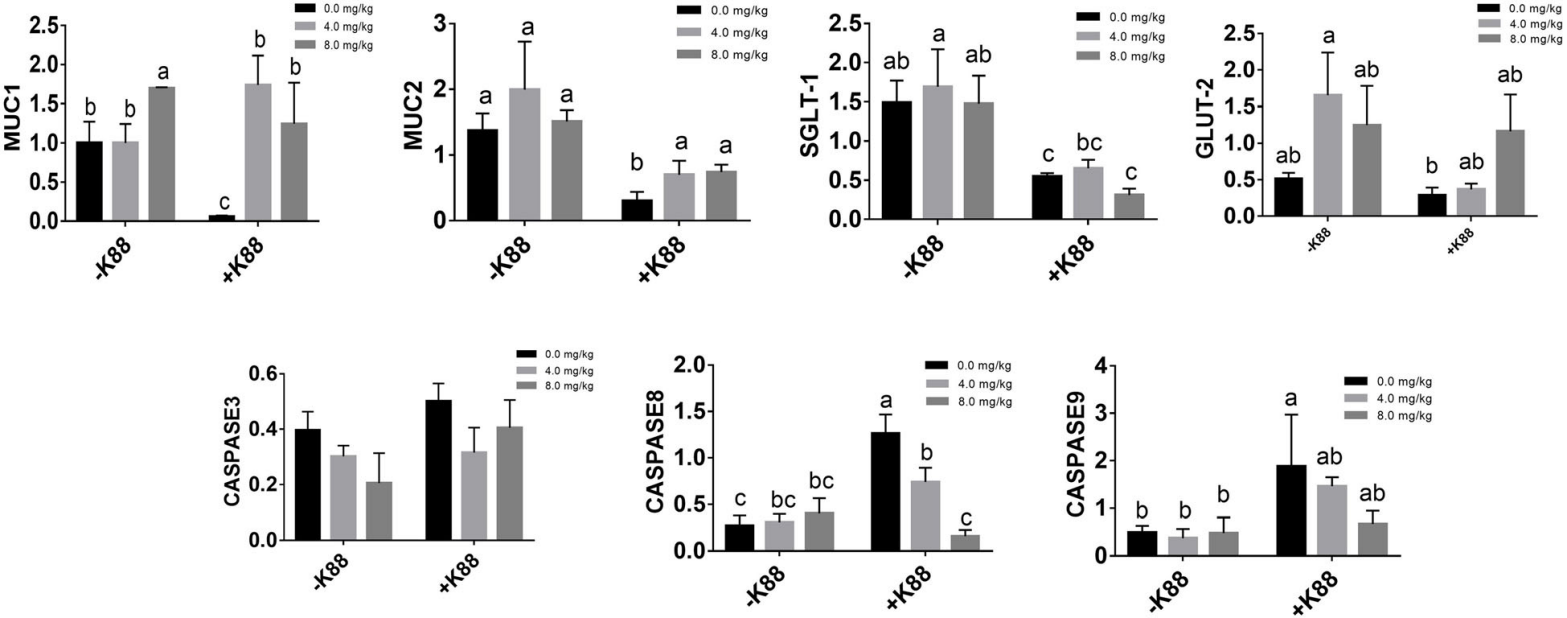
Effect of BMGlvA2 on the expression of critical genes related to intestinal barrier functions. Total RNA was extracted from jejunum tissue and the expression of related genes in jejunum was measured by real-time fluorescence PCR. The target gene mRNA expression level was calculated using the 2^–ΔΔCt^ method. ^a-c^ Values within a column differ if they do not share a common superscript (P < 0.05). MUC1: Mucin1; MUC2: Mucin2; SGLT-1: Sodium-dependent glucose transporter-1; GLUT-2: Glucose transporter-2; Caspase8, cysteinyl aspartate specific proteinase 8; Caspase9, cysteinyl aspartate specific proteinase 9

We also investigated the expression profiles of critical genes related to intestinal barrier functions. As shown in Fig. 5, ETEC challenge elevated the expression levels of two critical apoptosis related genes (caspase8 and caspase9), but significantly down-regulated the expression of genes related to epithelial functions such as the MUC1, MUC2, SGLT-1, and GLUT-2 (*P* < 0.05). However, BMGlvA2 treatment not only decreased the expression levels of caspase8, but also significantly elevated the expression levels of MUC1, MUC2, and GLUT-2 in the intestine (*P* < 0.05).

## Discussion

In recent years, the AMPs have attracted considerable research interest since they can serve as a substitute for conventionally used antibiotics or serve as a regulator to modulate the immunity [30]. Currently, a wide variety of forms of AMPs were isolated from mammals (including humans), insects, and amphibians. However, they share common properties such as antibacterial activity, broad spectrum, and nontoxic to mammalian cells [31, 32]. BMGlvA2 is a novel antibacterial peptide isolated from *B. mori*, which shows antibacterial activity against a broad microbial species [22]. In our previous study, the BMGlvA2 has been successfully expressed in *E. coli* and we found that the recombinant BMGlvA2 not only had significant antibacterial activities against both the gram-negative and positive bacteria (i.e. ETEC, *S. aureus*, and *B. subtilis*), but also had no hemolytic activity [27]. In this study, we explored the effect of BMGlvA2 in alleviating ETEC-induced inflammation and intestinal epithelium disruption in mice.

It is a well-known fact that ETEC infection not only induces severe diarrhea in animals, but also impairs a variety of tissues or organs [33]. The spleen is the largest lymphoid organ in the human body and plays a critical role in immune system functions [34]. In this study, ETEC challenge increased the fecal score (marking of the diarrhea) and impaired the spleen tissues. Moreover, the elevated serum concentrations of urea and crea indicated disruption of the kidney function in mice upon ETEC challenge [35, 36]. The elevated serum concentrations of IL-6 and TNF-α indicating an acute inflammatory response in mice upon ETEC challenge. These results indicated success of model construction. Interestingly, BMGlvA2 treatment significantly decreased the serum concentrations of IL-6 and TNF-α in ETEC-challenged mice, which suggested that the BMGlvA2 may act as a negative regulator for inflammatory responses. This result is consistent with previous studies on AMPs by using a variety of animal species [37–41]. It’s a fact that villi are critical components of the intestinal tract and their geometry provides an indicator of the absorptive capacity of the small intestine [42]. Villus height, crypt depth and the ratio of villus height to crypt depth (V/C) are common metrics for assessment of intestinal morphology [43]. The intestinal morphology can reveal some information on gut health. A shortening of the villus and deeper crypts may decrease the surface area of the intestinal tract for nutrient uptake. Studies of Gislason et al. and Swidsinski et al. found that the toxins produced by ETEC were closely related to the morphological changes of small intestine [44, 45]. In this study, ETEC challenge significantly decreased the villus height in the duodenum and jejunum, and decreased the V/C in the ileum. These results are similar to some previous studies ETEC challenge decreased the villus height and the V/C of the intestine [46–48]. However, BMGlvA2 treatment attenuated the ETEC-induced mucosa lesion. The tight junctions (TJs), which are composed of cytoplasmic scaffold proteins such as ZO-1, claudins, and attachment adhesion molecules, play a critical role in maintaining the intestinal permeability [49, 50]. However, various enteric pathogens can induce permeability defects in the intestinal epithelium by altering the distribution of tight junction proteins [51, 52]. Previous study indicated that ETEC infection elevated the mRNA and protein levels of tight junction proteins ZO-1 and occludin [53, 54]. In this study, the ZO-1 staining in the jejunum was diffuse with little staining at the intercellular tight junction region in the ETEC-challenged mice, indicating disruption of the TJs upon ETEC infection. However, BMGlvA2 treatment attenuated the ETEC-induced TJs disruption by improving the localization and abundance of the ZO-1 proteins. The improved mucosa morphology and tight junction by BMGlvA2 may be attributed to its antibacterial and anti-inflammatory activities, since the bacterial endotoxins (i.e. lipopolysaccharides) and inflammatory cytokines (i.e. TNF-α) are detrimental to the intestinal epithelium and both can induce the mucosa disruption [55–57].

To gain insights into the mechanisms behind the BMGlvA2 modulated intestinal barrier functions, we explored the expression levels of some critical molecules involved in the regulation of inflammatory response and apoptosis. Cytokines are an important part of the body’s cellular immune, which play a critical role in the development of lymphocyte and the subsequent functional activities of the peripheral immune compartment [58]. TNF-α, IL-1β and IL-6 are important Proinflammatory cytokine that regulate host immunity to a variety of pathogens through immune cell diferentiation, proliferation, and apoptosis [59]. However, excessive production of Proinflammatory cytokine might lead to body and gut damage [60]. As expected, ETEC challenge significantly elevated the expression levels of critical inflammatory response genes such as the IL-1β, IL-6, and TNF-α in the intestine, which was consistent with the previous reports [61, 62]. However, their expression levels were significantly down-regulated by BMGlvA2. The TLR4 and NF-κB are two critical signaling molecules involved in inflammation [63]. In this study, high dose BMGlvA2 treatment significantly decreased their expression levels in the intestine, which offers molecular basis for the BMGlvA2 modulated inflammatory responses. The caspase 8 and caspase 9 are two critical molecules responsible for executing cell death during the demolition phase of apoptosis [64]. MUC1 and MUC2 play important roles in maintaining intestinal epithelial barrier function [52]. In this study, BMGlvA2 significantly decreased the expression levels of caspase 8 and caspase 9, but increased the expression levels of genes related to intestinal barrier functions such as the MUC1, MUC2, and GLUT-2 in ETEC-challenged mice, indicating improved integrity of the intestinal epithelium by BMGlvA2.

## Conclusions

In conclusion, the BMGlvA2 attenuates ETEC-induced inflammatory responses and intestinal mucosa atrophy by reducing the secretion of inflammatory cytokines and the improving the morphology and integrity of intestinal epithelium. Our results suggested a novel function of the AMPs, and the anti-bacterial and anti-inflammatory properties of BMGlvA2 may allow it a potential agent to prevent or alleviate the inflammatory bowel diseases.

## Supplementary information

**Additional file 1: Figure S1.** SDS-PAGE analysis of rBMGlvA2 produced by *E. coli* Rosetta. *Lane 1* pET28a-Rosetta (induced), *Lane 2* pET32a-BMGlvA2-Rosetta (non-induced), *Lane 3–9* pET32a-BMGlvA2-Rosetta (Induction 3, 4, 5, 6, 7, 8, 9 h), M protein markers. **Table S1.** Primers for real-time PCR

## Data Availability

The datasets used and/or analysed during the current study are available from the corresponding author on reasonable request.

## Abbreviations

A/G: The ratio of albumin to globulin
ALB: Albumin
ALT: Alanine aminotransferase
AMPs: Antimicrobial peptides
AST: Glutinous straw transaminase
BMGlvA2: *Bombyx mori* gloverin A2
Caspase8: Cysteinyl aspartate specific proteinase 8
Caspase9: Cysteinyl aspartate specific proteinase 9
CREA: Creatinine
CRP: C-reactive protein
D-LA: D-lactic acid
ETEC: Enterotoxigenic *Escherichia coli*
GLB: Globulin
GLO: Globulin
GLUT-2: Glucose transporter-2
H&E: Hematoxylin and Eosin
ICR: Institute of Cancer Research
IL-1β: Interleukin 1 beta
IL-6: Interleukin 6
LPS: Iipopolysaccharides
MUC1: Mucin1
MUC2: Mucin2
NF-κB: Nuclear factor-kappa B
SGLT-1: Sodium-dependent glucose transporter-1
TLR4: Toll-like receptor 4
TNF-α: Tumor necrosis factor alpha
TP: Total protein
V/C: The ratio of villus height to crypt depth
ZO-1: Zonula occludens-1
