# Supplementary material for: Bombyx mori gloverin A2 alleviates enterotoxigenic Escherichia coli-induced inflammation and intestinal mucosa disruption
Source: Antimicrob Resist Infect Control. 2019 Nov 26;8:189. doi: 10.1186/s13756-019-0651-y (PMC6878672; doi:10.1186/s13756-019-0651-y)

**Table.S1 Primers for real-time PCR.**

| Gene | Primer Sequences | Product Size (bp) | GenBank Accession No. |
| --- | --- | --- | --- |
| ZO-1 | F: 5’- CGGTGCCCTGAAAGAAGCGA-3’ | 133 | BC138028.1 |
|  | R: 5’- ACCTGGGGCTGACAGGTAGG-3’ |  |  |
| Occludin | F: 5'-CCGGCCGCCAAGGTTC-3’ | 103 | [NM_008756.2](http://www.ncbi.nlm.nih.gov/entrez/viewer.fcgi?db=nucleotide&id=31982140) |
|  | R: 5’-ACTTTCAAAAGGCCTCACGGA-3’ |  |  |
| Claudin-2 | F: 5’-ATGGCATCCAGCAGAATACA-3’ | 131 | NM_016675 |
|  | R: 5’-ACCCACAAATGTACGGGAAT-3’ |  |  |
| SGLT-1 | F: 5’-GATTGGGGAACCACCCATGT3’ | 115 | [NM_0](https://www.ncbi.nlm.nih.gov/entrez/viewer.fcgi?db=nucleotide&id=133778943)19810.4 |
|  | R: 5’-AGCTTCAGTCCCTGCCTTTC-3’ |  |  |
| GLUT-2 | F: 5’-ACCGGGATGATTGGCATGTT-3’ | 150 | [NM_0](https://www.ncbi.nlm.nih.gov/entrez/viewer.fcgi?db=nucleotide&id=929981608)31197.2 |
|  | R: 5’-GGACCTGGCCCAATCTCAAA-3’ |  |  |
| Caspase9 | F: 5’-TCCTGGTACATCGAGACCTTG-3’ | 109 | NM_001355176.1 |
|  | R: 5’-AAGTCCCTTTCGCAGAAACAG-3’ |  |  |
| Caspase3 | F: 5’-ACAGCACCTGGTTACTATTC-3’ | 225 | NM_009810.3 |
|  | R: 5’-CAGTTCTTTCGTGAGCAT-3’ |  |  |
| IL-1β | F: 5’-ACCTGTGTCTTTCCCGTGG-3’ | 162 | NM_008361 |
|  | R: 5’-TCATCTCGGAGCCTGTAGTG-3’ |  |  |
| IL-6 | F:5’-AAAATTTCCTCTGGTCTTCTGGAGT-3’ | 154 | NM_001314054.1 |
|  | R:5’-TTCTGTGACTCCAGCTTATCTCTTG-3’ |  |  |
| TNF-α | F: 5’-TCTCATGCACCACCATCAAGGACT-3’ | 92 | [NM_013693.3](https://www.ncbi.nlm.nih.gov/entrez/viewer.fcgi?db=nucleotide&id=518831586) |
|  | R:5’-ACCACTCTCCCTTTGCAGAACTCA-3’ |  |  |
| β-actin | F: 5’-GCAAGCAGGAGTACGATGAGT-3’ | 86 | [NM_007393.5](https://www.ncbi.nlm.nih.gov/entrez/viewer.fcgi?db=nucleotide&id=930945786) |
|  | R: 5’-GGTGTAAAACGCAGCTCAGTA-3’ |  |  |
| NF-κB | F: 5’-CTGAGCGCCCCTCGCATTTA -3’ | 126 | NM_009045.5 |
|  | R: 5’-CCAGCCCATCTTTCTCAGCA-3’ |  |  |
| Caspase8 | F: 5’-CCACGGTGACAAGGGTGTCG-3’ | 185 | XM_006495634.3 |
|  | R: 5’-GTTGCTCGAAGCCTGCCTCA-3’ |  |  |
| TLR4 | F: 5’-TCTGGGGAGGCACATCTTCT-3’ | 112 | NM_021297.3 |
|  | R: 5’-TCAGGTCCAAGTTGCCGTTT-3’ |  |  |
| MUC1 | F: 5’-TCTTTCCAACCCAGGACACC-3’ | 128 | NM_013605.2 |
|  | R: 5’-ACTGCCATTACCTGCCGAAA-3’ |  |  |
| MUC2 | F: 5’-CTGCCCCAAGAAATGCCCCA-3’ | 185 | NM_023566.3 |
|  | R: 5’-GGCACTGGCTCACAGGGATG-3’ |  |  |

IL-1β: Interleukin 1 beta; IL-6: Interleukin 6; TNFα: Tumor necrosis factor alpha; ZO-1: Zonula occludens-1; MUC1: Mucin1; MUC2: Mucin2; GLUT-2: Glucose transporter-2; SGLT-1: Sodium-dependent glucose transporter-1; TLR4: Toll-like receptor 4; NF-κB:Nuclear factor-kappa B; Caspase3, Cysteinyl aspartate specific proteinase 3; Caspase8, Cysteinyl aspartate specific proteinase 8; Caspase9, Cysteinyl aspartate specific proteinase 9;

**Fig. S1 SDS-PAGE analysis of rBMGlvA2 produced by E. coli Rosetta.**


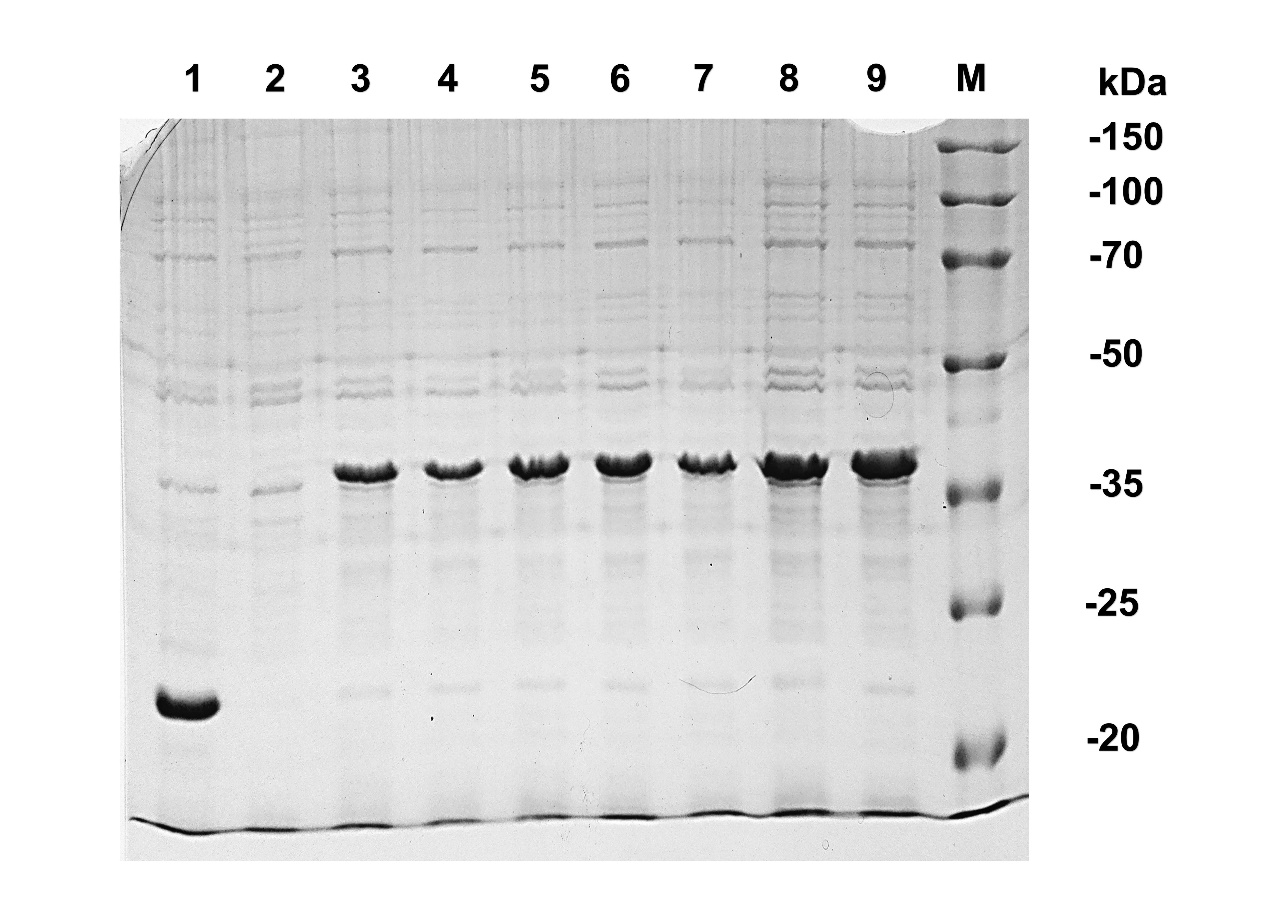

Supplement: Supplementary file 1 — Additional file 1: Figure S1. SDS-PAGE analysis of rBMGlvA2 produced by E. coli Rosetta. Lane 1 pET28a-Rosetta (induced), Lane 2 pET32a-BMGlvA2-Rosetta (non-induced), Lane 3–9 pET32a-BMGlvA2-Rosetta (Induction 3, 4, 5, 6, 7, 8, 9 h), M protein markers. Table S1. Primers for real-time PCR [file 13756_2019_651_MOESM1_ESM.docx]
